# Predictors of functioning in treatment-resistant schizophrenia: the role of negative symptoms and neurocognition

**DOI:** 10.3389/fpsyt.2024.1444843

**Published:** 2024-08-23

**Authors:** Yanhui Li, Mei San Ang, Jie Yin Yee, Yuen Mei See, Jimmy Lee

**Affiliations:** ^1^ North Region, Institute of Mental Health, Singapore, Singapore; ^2^ Research Division, Institute of Mental Health, Singapore, Singapore; ^3^ Department of Psychosis, Institute of Mental Health, Singapore, Singapore; ^4^ Lee Kong Chian School of Medicine, Nanyang Technological University, Singapore, Singapore

**Keywords:** treatment-resistant schizophrenia, functioning, negative symptoms, social anhedonia, neurocognition

## Abstract

**Introduction:**

Predictors of functioning are well-studied in schizophrenia, but much less so in treatment-resistant schizophrenia (TRS). In this study, we aim to investigate contributions of schizophrenia symptom domains and neurocognition to predict functioning in a TRS population (n = 146).

**Methods:**

Participants were assessed on the Positive and Negative Syndrome Scale (PANSS), to calculate scores for five symptom factors (Positive, Negative, Cognitive, Depressive and Hostility) and two negative symptom constructs (Diminished Expressivity (DE), and Social Anhedonia (SA) as part of the Motivation and Pleasure-related dimension), based on a previously validated model, modified in accordance with EPA guidelines on negative symptoms assessment. Neurocognition was assessed with symbol coding and digit sequencing tasks from the Brief Assessment of Cognition in Schizophrenia (BACS). Functioning was assessed with the Social and Occupational Functioning Assessment Scale (SOFAS), employment status and World Health Organization Disability Assessment Schedule 2.0 (WHODAS 2.0). Multiple regression analyses were performed on psychopathology scores and BACS scores against all three measures of functioning, controlling for age and sex. For WHODAS, regression with PANSS scores of significant symptom factors were also performed.

**Results:**

A lower severity of negative symptoms in the SA dimension was the strongest predictor of higher functioning across all three functioning measures. Neurocognition, in particular processing speed and attention assessed on the symbol coding task, predicted employment. A lower severity of somatic concerns and depressive symptoms was associated with lesser self-reported disability on WHODAS.

**Discussion:**

This study represents a first attempt at elucidating significant predictors of functioning in TRS. We highlight negative symptoms and neurocognition as important treatment targets to improve functioning in TRS, consistent with previous studies in general schizophrenia.

## 1 Introduction

Treatment-resistant schizophrenia (TRS) is defined as schizophrenia with a lack of response to 2 antipsychotic trials of adequate dose and duration (1), and makes up approximately 30% of all patients with schizophrenia (2). When compared to treatment-responsive schizophrenia, bipolar disorder, anxiety and depressive disorders, TRS patients were found to have the highest symptom severity, most severe cognitive impairment and poorest psychosocial functioning (3). Additionally, TRS contributes significantly to a reduction in quality of life and healthcare burden (4). Multiple studies have looked into predictors of functioning in patients with schizophrenia (5–7), but similar research for TRS remains scarce. Preliminary evidence suggests TRS could be a distinct clinical subtype as compared to treatment-responsive schizophrenia, with differing neurobiological causes and psychopathology (8). It is thus important to look into this subgroup separately, to shed light on significant factors we could mitigate, to improve overall function and quality of life. Studies in schizophrenia consistently highlight severity of negative symptoms and neurocognitive impairments as predictors of functioning (5–7). Negative symptoms constitute a core symptom cluster in schizophrenia, and can be conceptualised as five domains under two dimensions. The two overarching dimensions include deficits in expression, also known as diminished expression (DE), and deficits in motivation and pleasure (MAP) (9, 10). DE comprises the domains of Blunted affect and Alogia, while MAP comprises the domains of Anhedonia, Avolition, Asociality (11). Present studies have shown that a lower severity of negative symptoms, particularly in the MAP dimension, predicts better social and vocational functioning (5, 7, 10, 12–14). A higher severity of negative symptoms have been correlated with more severe neurocognitive deficits (15), but both negative symptoms and neurocognition may also affect function in schizophrenia independently. Negative symptoms may possibly mediate effects of neurocognition on function, but this is not well-established (16). Instead, general consensus focuses on the fact that neurocognition does affect various forms of functioning in schizophrenia (5–7). Different domains of neurocognition are associated with social or vocational functioning — processing speed and attention appear to be associated with both, while verbal memory was more specifically linked to psychosocial function (17). On the whole, executive function (18, 19), along with verbal learning and memory (18, 20) were found to be the most significant neurocognitive domains predicting employment in people with schizophrenia. These describe findings in general schizophrenia populations, and there are limited studies investigating TRS populations in this aspect. It is uncertain if similar trends apply to TRS, especially in regard to the significance of deficits in negative symptoms and MAP, since TRS is a clinical subtype more widely known for its enduring positive symptoms (21). In this study, we investigate the relative contributions of psychopathology via five symptom domains of schizophrenia and overall neurocognition, to predict functioning in TRS. We further investigate the relative contributions of the two negative symptom dimensions on functioning. We hypothesise that in TRS, a lower severity of negative symptoms, especially in the MAP-related dimension, and better neurocognition will predict higher functioning.

## 2 Materials and methods

### 2.1 Subjects

One hundred and fifty-nine individuals aged between 21 and 49, diagnosed with schizophrenia or schizoaffective disorder and on clozapine were recruited from inpatient wards and outpatient clinics at the Institute of Mental Health (IMH), Singapore. The diagnosis of schizophrenia/schizoaffective disorder was made in accordance with the DSM-IV criteria (22). Inclusion criteria consisted of patients currently prescribed clozapine with no changes to the current prescription for the past 2 weeks. All patients would have had at least two unsuccessful non-clozapine antipsychotic trials before being prescribed clozapine. Pregnant and lactating females were excluded from the study as ongoing pregnancy and lactation could affect cross-sectional functional assessments and employment status. Patients who lacked mental capacity to consent to the study were also excluded. Thirteen individuals had incomplete Social and Occupational Functioning Assessment Scale (SOFAS) assessments and were removed from subsequent analyses. Hence, total sample size available for this study is 146. All 146 subjects had complete data on SOFAS and employment status, but only 111 subjects managed to complete the World Health Organization Disability Assessment Schedule 2.0 (WHODAS 2.0). All procedures contributing to this work comply with the ethical standards of the relevant national and institutional committees on human experimentation and with the Helsinki Declaration of 1975, as revised in 2008. All procedures involving human subjects were approved by the National Healthcare Group’s Domain Specific Review Board (Approval Number 2015/00397). Written informed consent was obtained from all participants prior to joining this study.

Demographic data including age, sex, ethnicity, smoking status and marital status was obtained from study participants. Scores from the Clinical Global Impression-Severity Scale at point of recruitment were recorded (23). Past and current antipsychotic prescriptions were obtained from available medical records. Current antipsychotic doses were converted into chlorpromazine equivalent doses (24, 25).

### 2.2 Study assessments

#### 2.2.1 Psychopathology

Symptom severity was assessed on the Positive and Negative Syndromes Scale (PANSS) (26) by trained raters (intraclass correlation coefficient among raters = 0.80). To investigate individual symptom domains, we utilised a modified 5-factor PANSS model from exploratory factor analysis, previously validated in a multi-ethnic population (27). Scores from individual PANSS items were summed to obtain factor scores for the 5 symptom factors: Positive, Negative, Cognitive, Depressive and Hostility factors (**Supplementary Table 1**). In accordance with latest guidelines from the European Psychiatry Association recommending the inclusion of only core negative symptoms in assessment of the negative symptom dimension (28), we excluded PANSS Items of G7 Motor retardation and G16 Active social avoidance from the negative symptom factor and its two domains. The Negative symptom factor score was deconstructed into Diminished Expression (DE) and Social Anhedonia (SA) domains, as measures of deficits in expression and deficits in motivation and pleasure respectively. DE scores were calculated from summation of PANSS N1, N3 and N6 item scores while SA scores were calculated from summation of N2 and N4 item scores (27). These scores were used in subsequent analyses.

#### 2.2.2 Neurocognition

Participants were assessed on two tasks from the Brief Assessment of Cognition in Schizophrenia (BACS) (29) – symbol coding and digit sequencing. The former tests attention and processing speed while the latter tests working memory (29). Fervaha et al. (2014) had previously shown these two neurocognition domains contributed up to 76% of variance of global neurocognition in a large sample of schizophrenia patients (30). A recent meta-analysis also found the digit sequencing test to be one of the most sensitive in examining changes in cognitive functions in TRS (31). Hence, the symbol coding and digit sequencing tasks can be used as quick assessments for a good estimate of global neurocognition while minimising the need for labour-intensive administration of the full neuropsychological battery (30). Using data from local schizophrenia samples, we have established that these two tasks contributed 72% of variance of global neurocognition (unpublished results). Additionally, local patients tend to underperform on language-based tasks (32), so excluding language-based tasks may also avoid underestimation of neurocognition in our sample. Z-scores of the symbol coding and digit sequencing tasks against the distribution of normal population were recorded and averaged to obtain a composite neurocognitive z-score as a measure of global neurocognition (33). All three z-scores were used for subsequent analyses.

#### 2.2.3 Functioning

To comprehensively assess functioning, we utilised the Social and Occupational Functioning Assessment Scale (SOFAS) (34), employment status and the World Health Organization Disability Assessment Schedule 2.0 (WHODAS 2.0) (35). SOFAS measures social and occupational functioning independent of symptom severity and was administered by trained interviewers. A higher score on SOFAS indicates better functioning. Employment status was recorded as a binary measure of occupational functioning, i.e. employed or unemployed. Employment included full-time, part-time or sheltered work, while homemakers and students were considered unemployed. WHODAS 2.0 measures global functioning over six domains: Understanding and communicating, mobility, self-care, social and interpersonal functioning, home, academic and occupational functioning, as well as participation in society (35). The 36-item WHODAS 2.0 assessment was administered with trained interviewers interviewing participants in person; scores were computed through complex scoring using the online calculator provided by WHO (36). A lower WHODAS score indicates reduced disability and higher functioning.

### 2.3 Statistical analyses

All statistical analyses were carried out using IBM SPSS Statistics Version 29.0. Demographic data and clinical characteristics of the study population were analysed. In general, we performed univariate analyses, followed by multiple regressions for all 3 functioning outcome measures. Univariate regression was first employed to identify significant neurocognitive, or symptom factors associated with the functioning measure, to be included in subsequent multiple regression models. Variables with a *p* value ≤0.25 were considered significant and included in subsequent multiple regression models. A threshold of p ≤0.1- 0.25 has been suggested to be optimal in selecting variables via univariate analysis, to avoid being too lax or overconservative (37, 38). For each functioning measure, we employed 3 multiple regression models: first for significant symptom factors and/or neurocognitive composite identified from univariate analyses, then for DE and SA if PANSS negative factor was significant in the univariate analyses, and lastly for BACS symbol coding and digit sequencing if the neurocognitive composite was significant in the univariate analyses, to identify negative symptom subdomains and individual BACS tasks with the highest contribution to variance. All multiple regression models controlled for age and sex. Multicollinearity was assessed using Variance Inflation Factor and Tolerance in all regression analyses. Statistical significance was set at p <0.05 in the multiple regression analyses. In the analysis on WHODAS, two additional regression models were performed for the depressive and cognitive symptom factors as they were significantly associated with WHODAS. Individual PANSS items were entered in place of the depressive/cognitive factor scores, to identify underlying PANSS items driving the relationship of the symptom factors with WHODAS.

## 3 Results

### 3.1 Demographics and clinical characteristics of sample population

Demographics and clinical characteristics of the study sample are reported in **Table 1**. Mean PANSS total score for the study sample was 58.59 (*SD*=13.25). Mean scores from the Clinical Global Impression Severity Scale was 3.10 (Mildly ill, *SD*=1.24). The mean clozapine dose was 312.24 mg (*SD*=151.02; Range= 37.5 to 750) and 93 (63.7%) participants were on clozapine antipsychotic monotherapy.

**Table 1:** Demographics and clinical characteristics of study sample.

Variable	N (%)
Sex – Males (%)	93 (63.7)
Ethnicity	
Chinese (%)	125 (85.6)
Indian (%)	9 (6.20)
Malay (%)	7 (4.80)
Others (%)	5 (3.40)
Marital Status (Married, %)	15 (10.3)
Outpatients (%)	104 (71.2)
Smoking (Smoker, %)	20 (13.7)
Presence of psychiatric comorbidity (%)^a^	27 (18.5)
**Employment Status** (Employed, %)	60 (41.1)
Full Time	26 (43.3)
Part Time	24 (40.0)
Sheltered Employment	10 (16.7)
	Mean (S.D.)
Age (years)	39.1 (10.7)
Body Mass Index (kg/m^2^)	24.8 (4.8)
Duration of diagnosis (years)	15.5 (9.9)
Total Antipsychotic Dose (mg)^b^	377 (252)
SOFAS Score	59.9 (13.2)
WHODAS Score	19.0 (14.5)
Neurocognitive Composite Z-Score	-1.58 (1.07)
BACS Symbol Coding Z-Score	-1.76 (1.09)
BACS Digit Sequencing Z-Score	-1.40 (1.36)
Total PANSS score	58.6 (13.2)
Clinical Global Impression Severity Scale	3.10 (1.24)
Symptom Factors	
Positive	12.0 (5.39)
Negative	11.2 (4.37)
Cognitive^c^	12.4 (3.58)
Depressive	9.53 (3.61)
Hostility	4.92 (1.73)

^a^A maximum of 1 psychiatric comorbidity was present.

^b^In total daily Chlorpromazine equivalents.

^c^Missing data for 1 patient (N= 145).

### 3.2 Multiple linear regression on SOFAS

Neurocognitive composite (β=0.260, *t*=-3.229, *p*=0.002), positive (β=-0.311, *t*=-3.929, *p*<0.001), negative (β=-0.480, *t*=-6.569, *p*<0.001), cognitive (β=-0.327, *t*=-4.144, *p*<0.001), depressive (β=-0.127, *t*=-1.542, *p*=0.125) and hostility symptom factors (β=-0.146, *t*=-1.773, *p*=0.078) were identified as predictors of SOFAS in univariate linear regression to be included in the multiple regression models (**Table 2**). BACS symbol coding (β=0.301, *t*=3.792, *p*<0.001), BACS digit sequencing (β=0.174, *t*=2.125, *p*=0.035), DE (β=-0.334, *t*=-4.254, *p*<0.001) and SA (β=-0.507, *t*=-7.056, *p*<0.001) were also significant at the univariate level.

**Table 2:** Univariate and multiple linear regression on SOFAS.

Variables	Univariate analysis			Multiple Regression Model I			Multiple Regression Model II			Multiple Regression Model III		
	*β*	*t*	*p*	*β*	*t*	*p*	*β*	*t*	*p*	*β*	*t*	*p*
Neurocognitive Composite	0.260	3.229	**0.002**	0.047	0.582	0.561	0.037	0.463	0.644	–	–	–
BACS Symbol Coding	0.301	3.792	**<0.001**	-	-	-	-	-	-	0.173	1.793	0.075
BACS Digit Sequencing	0.174	2.125	0.035	-	-	-	-	-	-	-0.087	-0.962	0.338
Positive	-0.311	-3.929	**<0.001**	-0.241	-2.927	**0.004**	-0.186	-2.254	**0.026**	-0.221	-2.678	**0.008**
Negative	-0.480	-6.569	**<0.001**	-0.418	-5.294	**<0.001**	–	–	–	-0.404	-5.134	**<0.001**
DE	-0.334	-4.254	**<0.001**	-	-	-	-0.100	-1.205	0.230	-	-	-
SA	-0.507	-7.056	**<0.001**	-	-	-	-0.412	-5.028	**<0.001**	-	-	-
Cognitive	-0.327	-4.144	**<0.001**	-0.088	-1.013	0.313	-0.127	-1.478	0.142	-0.103	-1.171	0.244
Depressive	-0.127	-1.542	0.125	0.046	0.573	0.568	0.051	0.661	0.510	0.041	0.521	0.603
Hostility	-0.146	-1.773	0.078	-0.111	-1.459	0.147	-0.131	-1.752	0.082	-0.114	-1.499	0.136

DE, Diminished Expression; SA, Social Anhedonia.

BACS, Brief Assessment of Cognition in Schizophrenia.

Models adjusted for age and sex.

Model I parameters: R^2^ = 0.328, Adj. R^2^ = 0.288, F (8,136) = 8.286, p < 0.001.

Model II parameters: R^2^ = 0.364, Adj. R^2^ = 0.322, F (9,135) = 8.598, p < 0.001.

Model III parameters: R^2^ = 0.342, Adj. R^2^ = 0.298, F (9,135) = 7.790, p < 0.001.

P values < 0.05 are bolded.

In multiple regression, only positive (β=-0.241, *t*=-2.927, *p*=0.004) and negative symptom factors (β=-0.418, *t*=-5.294, *p*<0.001) remained significant predictors (Model I). Multiple linear regression looking into negative symptom dimensions revealed that lower severity of SA (β=-0.412, *t*=-5.028, *p*<0.001) and lower scores on positive symptoms (β=-0.186, *t*=-2.254, *p*=0.026) were significantly associated with higher SOFAS scores (Model II). DE was not a significant predictor (β=-0.100, *t*=-1.205, *p*=0.230). Although neurocognitive composite was a significant predictor of SOFAS in the univariate analysis, it was not significantly associated with SOFAS in the multiple regression models. Both BACS symbol coding (β=0.173, *t*=1.793, *p*=0.075) and BACS digit sequencing (β=-0.087, *t*=-0.962, *p*=0.338) were also not significantly associated with SOFAS in multiple regression (Model III).

### 3.3 Logistic regression on employment status

Neurocognitive composite (*OR*=2.056, CI=1.424-2.968, *p*<0.001), positive (*OR*=0.898, CI=839-0.960, *p*=0.002), negative (*OR*=0.845, CI=0.772-0.925, *p*<0.001), cognitive (*OR*=0.820, CI=0.731-0.920, *p*=0.001), depressive (*OR*=0.868, CI=0.783-0.963, *p*=0.007) and hostility symptom factors (*OR*=0.808, CI=0.639-1.021, *p*=0.075) were identified as predictors of employment status in univariate logistic regression to be included in subsequent multiple regression analyses (**Table 3**). BACS symbol coding (*OR*=2.060, CI=1.442-2.942, *p*<0.001) and BACS digit sequencing (*OR*=1.537, CI=1.167-2.025, p=0.002), DE (*OR*=0.852, CI=0.750-0.968, *p*=0.014) and SA (*OR*=0.700, CI=0.587-0.833, *p*<0.001) were also significant at the univariate level.

**Table 3:** Univariate and multiple logistic regression on employment status.

Variable	Univariate analysis			Multiple Regression Model IV			Multiple Regression Model V			Multiple Regression Model VI		
	Odds ratio	95% C.I	*p*	Odds ratio	95% C.I	*p*	Odds ratio	95% C.I	*p*	Odds ratio	95% C.I	*p*
Neurocognitive Composite	2.056	1.424-2.968	**<0.001**	1.548	1.022-2.345	**0.039**	1.509	0.997-2.316	0.054	–	–	–
BACS Symbol Coding	2.060	1.442-2.942	**<0.001**	-	-	-	-	-	-	1.045	1.003-1.088	**0.034**
BACS Digit Sequencing	1.537	1.167-2.025	**0.002**	-	-	-	-	-	-	1.014	0.919-1.119	0.777
Positive	0.898	0.839-0.960	**0.002**	0.953	0.878-1.035	0.256	0.967	0.888-1.054	0.450	0.960	0.883-1.044	0.340
Negative	0.845	0.772-0.925	**<0.001**	0.875	0.791-0.968	**0.010**	–	–	–	0.878	0.793-0.972	**0.012**
DE	0.852	0.750-0.968	**0.014**	-	-	-	0.995	0.849-1.166	0.952	-	-	-
SA	0.700	0.587-0.833	**<0.001**	-	-	-	0.728	0.588-0.901	**0.004**	-	-	-
Cognitive	0.820	0.731-0.920	**0.001**	0.960	0.834-1.105	0.574	0.931	0.802-1.080	0.343	0.954	0.827-1.099	0.512
Depressive	0.868	0.783-0.963	**0.007**	0.934	0.826-1.056	0.277	0.933	0.824-1.057	0.275	0.932	0.823-1.056	0.270
Hostility	0.808	0.639-1.021	0.075	0.864	0.664-1.125	0.278	0.858	0.664-1.110	0.244	0.856	0.655-1.119	0.255

DE, Diminished Expression; SA, Social Anhedonia.

BACS, Brief Assessment of Cognition in Schizophrenia.

Models adjusted for age and sex.

Model IV parameters: χ2 (df=8) = 35.698, p < 0.001.

Model V parameters: χ2 (df=9) = 39.600, p < 0.001.

Model VI parameters: χ2 (df=9) = 37.850, p < 0.001.

P values < 0.05 are bolded.

In multiple logistic regression, lower severity of negative symptoms (*OR*=0.875, CI=0.791-0.968, *p*=0.010) and better neurocognitive composite z-scores (*OR*=1.548, CI=1.022-2.345, *p*=0.039) were significantly associated with higher likelihood of being employed (Model IV). The remaining variables were not significantly associated with likelihood of employment. Logistic regression looking into dimensions of the negative symptom factor revealed a lower severity of SA (*OR*=0.728, CI=0.588-0.901, *p*=0.004) to be significantly associated with likelihood of employment, while DE was not (Model V). The third regression model with individual BACS tasks showed that higher BACS symbol coding z-score (*OR*=1.045, CI=1.003-1.088, *p*=0.034) and lower severity of negative symptoms (*OR*=0.878, CI=0.793-0.972, *p*=0.012) were associated with higher likelihood of being employed (Model VI).

### 3.4 Multiple linear regression on WHODAS

Univariate regression revealed neurocognitive composite (β=-0.133, *t*=-1.400, *p*=0.164), positive (β=0.206, *t*=2.202, *p*=0.030), negative (β=0.098, *t*=1.029, *p*=0.306), cognitive (β=0.190, *t*=2.007, *p*=0.047) and depressive (β=0.476, *t*=5.648, *p*<0.001) symptom factors as predictors for WHODAS to be included in the multiple regression analyses (**Table 4**).

**Table 4:** Univariate and multiple linear regression on WHODAS.

Variables	Univariate analysis			Multivariate analysis Model VII			Multivariate analysis Model VIII			Multivariate analysis Model IX		
	*β*	*t*	*p*	*β*	*t*	*p*	*β*	*t*	*p*	*β*	*t*	*p*
Neurocognitive Composite	-0.133	-1.400	0.164	0.015	0.154	0.878	0.031	0.330	0.742	–	–	–
BACS Symbol Coding	-0.079	-0.832	0.407	-	-	-	-	-	-	0.152	1.226	0.223
BACS Digit Sequencing	-0.143	-1.510	0.134	-	-	-	-	-	-	-0.074	-0.656	0.513
Positive	0.206	2.202	**0.030**	-0.042	-0.419	0.676	-0.109	-1.110	0.270	-0.016	-0.158	0.875
Negative	0.098	1.029	0.306	0.065	0.695	0.489	–	–	–	0.086	0.916	0.362
DE	-0.039	-0.411	0.682	-	-	-	-0.202	-2.001	**0.048**	-	-	-
SA	0.250	2.701	**0.008**	-	-	-	0.298	3.066	**0.003**	-	-	-
Cognitive	0.190	2.007	**0.047**	0.119	1.170	0.245	0.210	2.057	**0.042**	0.124	1.210	0.229
Depressive	0.476	5.648	**<0.001**	0.476	4.911	**<0.001**	0.446	4.762	**<0.001**	0.468	4.816	**<0.001**
Hostility	0.035	0.368	0.714	–	–	–	–	–	–	–	–	–

DE, Diminished Expression; SA, Social Anhedonia.

BACS, Brief Assessment of Cognition in Schizophrenia.

Models adjusted for age and sex.

Model VII parameters: R^2^ = 0.246, Adj. R^2^ = 0.194, F (7,102) = 4.741, p < 0.001.

Model VIII parameters: R^2^ = 0.310, Adj. R^2^ = 0.256, F (8,101) = 5.679, p < 0.001.

Model IX parameters: R^2^ = 0.256, Adj. R^2^= 0.198, F (8,101) = 4.353, p < 0.001.

P values < 0.05 are bolded.

In multiple linear regression of significant symptom factors against WHODAS, depressive symptom factor was consistently positively associated with WHODAS across the three models (Models VII, VIII, IX). When negative symptom dimensions of DE and SA were entered in place of the negative symptom factor, both were significantly associated with WHODAS (DE: β=-0.202, *t*=-2.001, *p*=0.048; SA: β=0.298, *t*=3.066, *p*=0.003), along with the cognitive (β=0.210, *t*=2.057, *p*=0.042) and depressive symptom factors (β=0.446, *t*=4.762, *p*<0.001) (Model VIII). A higher severity of DE, lower severity of SA, and less severe scores on cognitive and depressive symptom factors, were associated with lower disability scores and better functioning. When scores of individual BACS tasks were entered in place of the neurocognitive composite z-score, both scores of individual BACS tasks were not significantly associated with WHODAS (Model IX).

The depressive symptom factor consisted of multiple PANSS items measuring more than just depressive symptoms. To investigate symptoms underlying the depressive symptom factor’s association with WHODAS, we entered individual PANSS items in place of the depressive symptom factor in multiple regression (**Supplementary Table 2**: Model X). Somatic Concern (G1) (β=0.264, *t*=2.855, *p*=0.005) and Depression (G6) (β=0.232, *t*=2.028, *p*=0.045) were found to contribute a significant amount of variance in predicting WHODAS. A lower severity of somatic concern and depressive symptoms were associated with lower disability and higher functioning. All other PANSS items constituting the depressive symptom factor were not significantly associated with WHODAS in multiple regression. Individual PANSS items constituting the cognitive symptom factor were also examined (Model XI). Higher scores on difficulty in abstract thinking (N5) (β=-0.268, *t*=-2.529, *p*=0.013) were associated with lower WHODAS disability and higher functioning. A significant but small inverse correlation was found between PANSS Lack of judgment and insight (G12) and WHODAS scores (*r_s_* = -0.281, *p*= 0.003), suggesting an association between poor insight and judgement with lower self-rated disability scores and higher self-rated functioning. PANSS difficulty in abstract thinking (N5) was also significantly positively correlated with PANSS Lack of judgment and insight (G12) (*r_s_* = 0.291, *p*<0.001), and inversely correlated with WHODAS (*r_s_* = -0.276, *p*=0.003). This suggests patients with increased difficulty in abstract thinking had poorer insight and rated themselves lower on WHODAS.

## 4 Discussion

### 4.1 Main findings

In the present study on TRS, we have shown that a lower severity in the SA dimension of negative symptoms significantly predicted better functioning across all three indicators − SOFAS, WHODAS and employment status. The positive symptom factor was also predictive of higher SOFAS score. Better cognitive performance predicted a higher likelihood of employment particularly through processing speed and attention as assessed by the symbol coding task. Better cognitive performance was not associated with SOFAS nor WHODAS in our sample. Additionally, a lower severity of somatic concerns and depressive symptoms were associated with lower self-rated disability and higher functioning, as measured through the WHODAS. Lower scores on difficulty in abstract thinking were associated with lower WHODAS disability scores and higher functioning. **Supplementary Table 3** summarizes significant predictors of functioning across regression models on all three functioning outcome measures.

### 4.2 Comparison with findings from other studies

To the best of our knowledge, this is the first study looking into predictors of global, social and vocational measures of functioning in TRS simultaneously. Our findings corroborate what has been shown for schizophrenia in general: a lower severity of negative symptoms, especially in the motivation and pleasure dimension, predicted better functioning (5, 7, 14). Yang et al. (2021) had shown that after accounting for neurocognition and functional capacity, negative symptoms contributed additional variance to predicting real world functioning in a schizophrenia population. Harvey et al. (2017) reported that negative symptoms, specifically avolition-apathy, predicted social function better than DE. Avolition-apathy was also previously associated with a higher degree of impairment of social and vocational function in schizophrenia, as compared to DE (10, 12). No studies have looked into predictors of functioning measures in TRS, but we note one recent study by Iasevoli et al. (2018) looking into predictors of functional capacity in TRS (39). It is prudent to note that functional capacity cannot be equated to real world functioning. The former relies more on neurocognitive abilities within controlled environments, while the latter is more complex and involves integration of symptomatology and social functioning (40). Nevertheless, Iasevoli et al. (2018) found the PANSS negative score to be the strongest predictor of functional capacity, contributing to the largest variance.

For employment, negative symptoms, especially deficits in the motivation and pleasure dimension, along with neurocognition have been found to predict employment status or vocational function (5, 12, 41). Kaneda et al. (2010) previously investigated predictors of employment status after 12 months of treatment with clozapine for TRS (42). They found cognition, specifically verbal working memory, to be the only significant predictor of employment status; severity of positive and negative symptoms were not significant predictors. However, these findings may have been limited by the small sample size of 59 patients. Other previously reported predictors of employment in schizophrenia include social support and skills, past employment history (43), psychosocial rehabilitation (44), fatalistic control beliefs (41), the amount of government entitlement income received and engagement in sheltered work activity (18). Of note, cognition was found to predict work performance most strongly (45) while prior employment history was the most consistent predictor among studies (18, 19, 43). Of multiple neurocognitive domains, executive function (18, 19), along with verbal learning and memory (18, 20) were found to be the most significant predictors of work outcomes in people with schizophrenia. Interestingly, positive symptoms were mostly not predictive of employment in past studies (44, 46), a finding we found in TRS as well.

The significance of neurocognition, specifically processing speed and attention as assessed by the symbol coding task, in predicting employment status is corroborated by current literature. Milev et al. (2005) showed verbal memory, processing speed and attention domains to be significant predictors of global psychosocial function and recreational impairment in schizophrenia (17). However, only processing speed and attention were significant predictors of work impairment while verbal memory was significant for predicting relationship impairments. Our finding supports the important role of processing speed and attention in maintaining good vocational function.

### 4.3 Findings on WHODAS

A large number of findings on WHODAS likely stem from it being a self-reported scale (35) as opposed to SOFAS which is interviewer-rated. Our study found that patients with higher PANSS depressive scores rated themselves as being much more impaired on the WHODAS. Within the depressive symptom factor, PANSS somatic concerns (G1) and PANSS depression (G6) items were positively associated with WHODAS. Patients with increased somatic concerns have been found to be more self-absorbed (47), possibly explaining the association with higher self-reported disability scores. Disability scores have also been found to be strongly associated with severity of depressive symptoms and quality of well-being, but not with cognition and everyday functioning (48, 49). Higher severity of depression has been shown to be associated with increased self-awareness of disability (50, 51), so one would expect higher self-rated disability scores, in line with what we have found. The impact of mood on other self-reported scales such as quality-of-life scales have been reported (52), and higher levels of mood and anxiety problems have also been associated with increased self-reported disability with WHODAS (53). Patients with poorer insight as assessed by the PANSS G12 item also rated themselves lower on disability scores. Our results suggest patients with poorer mood and higher awareness of their own psychiatric condition tend to rate themselves more poorly on disability scores.

In our analysis on WHODAS, the cognitive symptom factor was positively associated with WHODAS although the neurocognitive composite was not. This is not contradictory as the cognitive symptom factor measures more than just global neurocognition – it constitutes seven PANSS items including: poor attention, conceptual disorganisation, stereotyped thinking, difficulty in abstract thinking, disturbance of volition, mannerism and posturing and preoccupation (**Supplementary Table 1**). On examination of PANSS items of the cognitive symptom factor, N5 Difficulty in abstract thinking was inversely associated with WHODAS. Given that PANSS difficulty in abstract thinking (N5) was significantly positively correlated with PANSS Lack of judgment and insight (G12) but inversely correlated with WHODAS, it seems patients with increased difficulty in abstract thinking tended to have poorer insight, and tended to rate themselves lower on WHODAS. If not for the self-reported nature of WHODAS, we would expect a positive association between difficulty in abstract thinking and disability scores, as previous studies have found worse abstract thinking to be associated with poorer function (54).

We also found a lower severity of SA but higher severity of DE to be associated with lower self-reported disability scores. Negative symptoms have been shown to strongly predict disability (55), though no previous studies have looked into specific associations of SA and DE subdomains with disability scores. The former association is more intuitive, with the latter negative association between DE and disability scores being less so. DE has been shown to be less psychopathologically severe than SA and is associated with better overall function (10). Patients with more severe expressivity symptoms may be less perturbed by the symptoms and less prone to expressing how they have been negatively impacted, hence rating themselves lower on disability scores. However, there is a caveat in interpreting this result as there is a possible suppressor effect, with SA influencing the relationship between DE and WHODAS. Hierarchical regression with sequential addition of variables into the model showed that addition of SA resulted in DE becoming significantly associated with WHODAS (*p* = 0.008 from *p*=0.316; increase in adjusted R^2^ from 0.198 to 0.305). DE is significantly correlated with SA (R_s_ = 0.454, *p*<0.001), while SA is positively correlated with WHODAS (R_s_ = 0.266, *p*=0.005). DE itself is not significantly correlated with WHODAS. Several studies have shown a moderate association between social anhedonia and diminished expressivity symptoms (11, 56). Further studies into this relationship would be prudent to control for it in future.

### 4.4 Discrepancies in findings across the three measures of functioning

Discrepancies in significant predictors across regression analyses for SOFAS, employment status and WHODAS are likely related to the fact that these outcomes measure different aspects of functioning. We did not find neurocognition to be a significant predictor of SOFAS or WHODAS, but it was a significant predictor of employment status. This could be related to the specific aspect of functioning which neurocognition affects. Strassnig et al. (2015) had previously shown that cognition affected everyday and vocational functioning specifically, but did not significantly impact social functioning (14). A lower severity of positive symptoms was found to be associated with higher functioning in SOFAS, but was not associated with employment status and WHODAS scores. Previous studies in schizophrenia found both positive and negative symptoms to be associated with functioning (11, 57), and most did not find positive symptoms to be predictive of employment (44, 46, 58). SOFAS contains measures of psychosocial functioning while employment status reflects purely vocational functioning. Our findings raises the possibility that positive symptoms may have a greater impact on psychosocial functioning compared to vocational functioning.

### 4.5 Limitations

One notable limitation in our study is the use of PANSS to evaluate negative symptoms dimensions. While PANSS is widely adopted and has previously been validated in assessment of negative symptom dimensions (59), it lacks specific items to assess experiential deficits comprehensively. Not all motivation and pleasure (MAP)-related symptoms are able to be measured in this study and results on SA relating to the MAP dimension may be underestimated. Secondly, our study does not differentiate between primary and secondary negative symptoms. Secondary negative symptoms may arise as a consequence of positive symptoms (such as social withdrawal due to persecutory delusions), comorbid depressive symptoms or social isolation from lack of social support from relatives or prolonged hospitalization (60). Although the impact of positive and depressive symptoms on functioning outcomes were adjusted for, other aspects such as poor social support or prolonged hospitalisation were not explored. We are also unable to account for side effects of medications and duration of illness which could impact functional outcomes. Medication side effects could possibly contribute to secondary negative symptoms. Thirdly, our study is also limited by the exclusion of other domains of neurocognition, given that we have utilised only two BACS tasks assessing working memory, attention and processing speed. Although these two domains give a good indication of overall cognitive function (30), we are unable to evaluate the contributions of other relevant cognitive domains, such as verbal memory and semantic fluency, to functioning in TRS. Although our study population consisted entirely of TRS patients, majority (71.2%) of them were outpatients with relatively well-controlled psychotic symptoms (mean PANSS score = 58.6, S*D* = 13.2). Further studies are required to corroborate our findings in TRS populations with more severe and poorly-controlled psychotic symptoms. We acknowledge the heterogeneity of the TRS population (61, 62), and future studies may also perform subgroup analysis to investigate possible different findings in clozapine-responsive versus clozapine-resistant patients. We did not attempt this as our study was not designed to measure clear clozapine response statuses, and we had a limited sample size for subgroup analysis. Lastly, we performed a cross-sectional study and a longitudinal study would be useful in future, to investigate relative contributions of negative symptoms and neurocognition on functioning over the course of the illness trajectory. This can guide interventions targeting the appropriate domains at specific points in time to improve function.

## 5 Conclusions

In conclusion, our findings highlight negative symptoms, in particular deficits in motivation and pleasure, and neurocognition as vital treatment targets to improve functioning in individuals with TRS. Further studies are required to explore possible interventions to this end, and in elucidating the trajectory of decline in these domains over time. Lastly, clinicians are reminded to monitor and manage somatic concerns and depressive symptoms which were associated with patients’ perceived disability and poorer self-reported functional outcomes.

## Data Availability

The datasets presented in this article are not readily available because the participants of this study did not agree for their data to be shared publicly, so supporting data is not publicly available. Requests to access the datasets should be directed to imhresearch@imh.com.sg.
