# Supplementary material for: Predictors of functioning in treatment-resistant schizophrenia: the role of negative symptoms and neurocognition
Source: Front Psychiatry. 2024 Aug 23;15:1444843. doi: 10.3389/fpsyt.2024.1444843 (PMC11411185; doi:10.3389/fpsyt.2024.1444843)
Supplement: Supplementary file 1 [file Table1.docx]

**Supplementary Table 1. Eclectic model of PANSS items comprising five clinical symptom factors**

| Number | PANSS Item | Clinical symptom factor |
| --- | --- | --- |
| 1 | P1 Delusions | Positive symptom factor |
| 2 | P3 Hallucinatory behaviour |  |
| 3 | P5 Grandiosity |  |
| 4 | P6 Suspiciousness/persecution |  |
| 5 | G9 Unusual thought content |  |
| 6 | N1 Blunted affect | Negative symptom factor |
| 7 | N2 Emotional withdrawal |  |
| 8 | N3 Poor rapport |  |
| 9 | N4 Passive/apathetic social withdrawal |  |
| 10 | N6 Lack of spontaneity and flow of conversation |  |
| 11 | P2 Conceptual disorganisation | Cognitive symptom factor |
| 12 | N5 Difficulty in abstract thinking |  |
| 13 | N7 Stereotyped thinking |  |
| 14 | G5 Mannerism & posturing |  |
| 15 | G11 Poor attention |  |
| 16 | G13 Disturbance of volition |  |
| 17 | G15 Preoccupation |  |
| 18 | G1 Somatic concern | Depressive symptom factor |
| 19 | G2 Anxiety |  |
| 20 | G3 Guilt feelings |  |
| 21 | G4 Tension |  |
| 22 | G6 Depression |  |
| 23 | P4 Excitement | Hostility symptom factor |
| 24 | P7 Hostility |  |
| 25 | G8 Uncooperativeness |  |
| 26 | G14 Poor impulse control |  |
| 27 | G10 Disorientation | Not applicable |
| 28 | G12 Lack of judgment and insight | Not applicable |
| 29 | G7 Motor retardation | Not applicable |
| 30 | G16 Active social avoidance | Not applicable |

**Supplementary Table 2. Multiple linear regression on WHODAS for PANSS items constituting the depressive and cognitive symptom factors**

| Variables | Multivariate analysis  Model X | | | Multivariate analysis  Model XI | | |
| --- | --- | --- | --- | --- | --- | --- |
|  | *β* | *t* | *p* | *β* | *t* | *p* |
| Neurocognitive Composite | 0.016 | 0.163 | 0.871 | -0.083 | -0.816 | 0.416 |
| Positive | -0.074 | -0.705 | 0.483 | -0.054 | -0.543 | 0.588 |
| Negative | 0.082 | 0.831 | 0.408 | 0.108 | 1.144 | 0.256 |
| Cognitive | 0.115 | 1.064 | 0.290 | - | - | - |
| P2 Conceptual disorganisation | - | - | - | 0.147 | 1.541 | 0.127 |
| N5 Difficulty in abstract thinking | - | - | - | -0.268 | -2.529 | **0.013** |
| N7 Stereotyped thinking | - | - | - | 0.069 | 0.647 | 0.519 |
| G5 Mannerism & posturing | - | - | - | 0.065 | 0.738 | 0.462 |
| G11 Poor attention | - | - | - | 0.047 | 0.441 | 0.660 |
| G13 Disturbance of volition | - | - | - | 0.141 | 1.535 | 0.128 |
| G15 Preoccupation | - | - | - | -0.004 | -0.034 | 0.973 |
| Depressive | - | - | - | 0.336 | 3.244 | **0.002** |
| G1 Somatic concern | 0.264 | 2.855 | **0.005** | - | - | - |
| G2 Anxiety | 0.213 | 1.827 | 0.071 | - | - | - |
| G3 Guilt feelings | 0.059 | 0.608 | 0.545 | - | - | - |
| G4 Tension | -0.030 | -0.326 | 0.745 | - | - | - |
| G6 Depression | 0.232 | 2.028 | **0.045** | - | - | - |
| Hostility | - | - | - | - | - | - |

Models adjusted for age and sex.

Model X parameters: R^2^= 0.277, Adj. R^2^= 0.196, *F (11,98)* = 3.418, p < 0.001

Model XI parameters: R^2^= 0.335, Adj. R^2^= 0.245, *F (13,96)* = 3.724, p < 0.001

P values < 0.05 are bolded.

**Supplementary Table 3. Summary of significant predictors of functioning across multiple regression models on SOFAS, employment status and WHODAS**

|  | SOFAS | | | Employment status | | | WHODAS | | |
| --- | --- | --- | --- | --- | --- | --- | --- | --- | --- |
| Variable | Model  I | Model  II | Model  III | Model  IV | Model  V | Model  VI | Model  VII | Model VIII | Model  IX |
| Neurocognitive Composite | No | No | - | Yes | No | - | No | No | - |
| BACS Symbol Coding | - | - | No | - | - | Yes | - | - | No |
| BACS Digit Sequencing | - | - | No | - | - | No | - | - | No |
| Positive | Yes | Yes | Yes | No | No | No | No | No | No |
| Negative | Yes | - | Yes | Yes | - | Yes | No | - | No |
| DE | - | No | - | - | No | - | - | Yes | - |
| SA | - | Yes | - | - | Yes | - | - | Yes | - |
| Cognitive | No | No | No | No | No | No | No | Yes | No |
| Depressive | No | No | No | No | No | No | Yes | Yes | Yes |
| Hostility | No | No | No | No | No | No | - | - | - |

*DE, Diminished Expression; SA, Social Anhedonia*

*BACS, Brief Assessment of Cognition in Schizophrenia*

Statistical significance in multiple regression: p≤0.05.
